# 非小细胞肺癌*EGFR*突变的检测方法

**DOI:** 10.3779/j.issn.1009-3419.2011.03.18

**Published:** 2011-03-20

**Authors:** 鑫宇 张, 皈阳 刘, 晓光 祝, 伟兰 王

**Affiliations:** 1 233000 蚌埠，蚌埠医学院药学系 Department of Pharmacy, Bengbu Medical College, Bengbu 233000, China; 2 100037 北京，解放军总医院第一附属医院药剂药理科 Department of Pharmacy, the Affiliated Hospital of Chinese PLA Hospital, Beijing 100037, China; 3 100853 北京，解放军总医院药品保障中心 Department of Pharmaceutical Care, PLA General Hospital, Beijing 100853, China

非小细胞肺癌（non-small cell lung cancer, NSCLC）是严重危害人类健康的常见恶性肿瘤。近年来表皮生长因子受体酪氨酸激酶抑制剂（epidermal growth factor receptor-tyrosine kinase inhibitor, EGFR-TKI）为NSCLC的治疗带来新的曙光，但用药前必须依据表皮生长因子受体（epidermal growth factor receptor, EGFR）的突变检测结果选择治疗对象^[1-4]^。因此*EGFR*突变成为肺癌患者应用TKI有无疗效的关键因素。探索和建立快速准确检测NSCLC患者*EGFR*基因突变的方法，有助于筛选出适合治疗的人群，具有较好的临床意义。从目前收集到的资料来看，*EGFR*突变检测的方法多种多样，主要有PCR（polymerase chain rection）-直接测序法^[5]^、PCR-TaqMan法^[6-9]^、变性高效液相色谱法（denaturing high-performance liquid chromatography, DHPLC）^[10-12]^、蝎形探针扩增阻滞突变系统法（scor pions amplification refractory mutation system, SARMS）^[6, 13-19]^、聚合酶链式反应-单链构象多态性（polymerase chain reaction-single strand conformation polymorphism, PCR-SSCP）法^[16, 20]^、酶切富集PCR法^[21-23]^及聚合酶链式反应连接的限制性片段长度多态性分析（polymerase chain reaction-restriction-fragment length polymorphism, PCR-RFLP）法^[24]^等^[25, 26]^。奉水东等^[27]^2008年针对肺癌*EGFR*基因突变检测方法进行过讨论，但未对各种方法适用范围进行详细比较，加之近几年又出现了一些新的技术方法，尤为表现在检测样本的范围拓展方面。因此本文就上述方法进行综述。

## 1 各种方法检测原理

无论是哪一种方法，首先都要进行样本DNA的提取，样本类型主要包括手术切除或CT引导下穿刺获取的肿瘤组织、癌性胸腔积液以及外周血等。为得到足量、稳定、均一的DNA样本，推荐采用DNA快速抽提纯化试剂盒进行提取纯化然后进行扩增检测。

### 1.1 PCR-直接测序法

根据文献^[1, 2]^报道的方法，应用PCR直接扩增*EGFR*基因第18、19、20、21的基因片段。扩增片段同时包含外显子及其相连的内含子部分。PCR反应条件为变性95 ℃、15 min；变性94 ℃、30 s，退火65 ℃、30 s和延伸72 ℃、1 min，35个循环；最后延伸72 ℃、5 min。2%琼脂糖凝胶电泳鉴定扩增片段，通过凝胶成像系统观察PCR产物电泳结果。PCR产物进行正反两个方向测序。分析测序图谱，判断*EGFR*基因第18、19、20、2l外显子突变区域是*EGFR*突变检测的金标准。

### 1.2 PCR-TaqMan法

根据文献^[1, 2]^报道的方法，设计针对*EGFR*基因19外显子4个缺失区域的检测探针（突变型探针）、针对第21外显子L858R突变和18外显子G719C点突变检测探针；同时设计相应的基因野生型检测探针。探针的5′端采用FAM、TET和HEX荧光染料进行标记，3′端与非荧光淬灭分子（minor groove binder, MGB）相连接。使用Lightcylcer PCR和反应体系进行实时PCR扩增，PCR条件为94 ℃变性3 min，94 ℃、5 s，60 ℃、30 s，循环50次。530 nm下实时监测产物。

MGB技术则利用与双链DNA的小沟的高度亲和力增加寡核苷酸探针结合到单链模板DNA上的稳定性，提高探针Tm值，缩短了探针的设计长度，增加配对与非配对时Tm上的差异，改善探针的特异性与敏感性，对单核苷酸突变检测更为有效。

### 1.3 DHPLC

DHPLC检测使用WAVE4500核苷酸分析系统，色谱柱为DNA SepCartridge分离柱，流动相为不同浓度的乙腈与三乙基铵醋酸盐（triethylamine acetate, TEAA）配制的洗脱液，由控制软件WAVE Maker根据待测DNA序列自动生成乙腈梯度和柱温，以0.9 mL/min流速测定，PCR产物不作任何纯化处理，直接用于DHPLC分析。19外显子突变类型为15 bp-18 bp碱基删除突变，采用非变性条件在50 ℃条件下对片段长度进行测定，野生型产物为151 bp，删除突变产物由于片段较短（136 bp-133 bp）会先于野生型产物出现而与野生型峰明显分离。外显子21的突变类型为L858置换突变采用部分变性温度进行测定，先将PCR产物变性复性处理，若存在突变会形成杂合双链，其退火温度低于纯和双链，61 ℃部分变性条件下可将产物分离。将测序阳性的标本作为阳性对照，以注射用水作为空白对照。

### 1.4 SARMS

ARMS（amplification refractory mutation system）扩增阻滞突变系统技术用于等位基因的鉴定，附加错配突变设计在3′端引物序列中，从而实现特异性的扩增。Scorpions为蝎形结构的特异性探针，包含一个与3′端共价相连的PCR引物，引物仅仅识别突变序列，而不识别正常序列，在即时PCR反应中，当探针与扩增子（即突变序列）连接进行扩增时，荧光基团与淬灭基团分离，反应试剂中荧光度增强。Scorpions与ARMS技术联和应用（SARMS）可检测单个突变，对于已知基因的检测，SARMS具有高度的敏感性^[13, 28, 29]^。

现今英国的Delivering Pharmacogenomics公司推出的EGFR突变检测试剂盒（即EGFR Scorpion Kit；DxS Ltd. Manchester，英国），可检测EGFR外显子18、19、20、21四个外显子的29种体细胞突变。

### 1.5 PCR-SSCP

PCR-SSCP^[20]^是一种经典的检测基因突变的方法，原理为：单链DNA因链内碱基配对而具有一定的空间构象，当DNA链上的碱基发生改变时，单链DNA会形成不同的构象，即单链构象多态性。在非变性聚丙烯酰胺凝胶电泳中，不同构象的DNA分子具有不同的泳动速度。单链DNA分子的相对迁移率不仅与DNA分子的大小有关，而且与其构象有关。

### 1.6 酶切富集PCR法

酶切富集PCR法是进行核酸扩增的有效方法。该方法基于已知的突变类型设计相应的引物，然后根据PCR产物的性质（有无或大小）来判断特异性突变的存在。一般分两步进行，首先利用限制性内切酶去选择性消化野生型的*EGFR*基因片断，从而使反应体系中突变型*EGFR*基因片断得到富集，再通过PCR放大和凝胶电泳来进行突变的判断。

### 1.7 PCR-RFLP

PCR-RFLP是在PCR和DNA序列分析基础上产生的RFLP技术。该方法是通过PCR扩增一段DNA片段，然后再选择适当的限制性内切酶，消化PCR产物，经电泳可得到有特异性的电泳谱带，从而达到鉴定不同基因型的目的。

### 1.8 其它

#### 1.8.1 核酸肽锁核酸聚合酶链式反应^[25]^（peptide nucleic acid-locked nucleic acid.PNA-LNA）

PNA-LNA检测*EGFR*基因已知位点的突变。含有野生序列的核酸肽（PNA）和含有突变探针的锁核酸（LNA）被使用去构建PCR夹，因为PNA引物只能结合在野生序列上而不能与突变序列结合，从而起到了选择性地阻止PCR引物扩增野生序列的作用，即“锁”的功能。该法降低了对检测组织的要求，大大提高了突变检测的灵敏度。

#### 1.8.2 轻链PCR^[26]^

轻链PCR方法采用探针杂交方式进行DNA检测和定量的荧光PCR技术。在反应成分中，除了常规PCR的反应成分外，还包括两条标记有荧光基团的寡聚核苷酸探针，这两条寡聚核苷酸探针均与DNA模板的碱基配对，而且其中一条探针的3′端标记了荧光基团（荧光素），而另一条探针则在5′端标记了荧光基团，当两个基团之间只有1个-5个碱基的距离时，便发生了高效的荧光共振能量转移。在PCR反应进行到退火步骤时，两条寡聚核苷酸探针与DNA模板杂交，第一条探针的荧光基团（荧光素）受到光源的照射发出一种绿色荧光，由于两个荧光基团的距离相当小，因此便发生了能量转移，从而激发了另一个荧光基团发出更强的红色荧光信号，当DNA模板量增加时，荧光基团发出的荧光信号也随之增加，因此在退火阶段检测的荧光信号的强度便可以检测相应的DNA模板数量，实现了目的基因的定量检测。

## 2 方法之间的比较

### 2.1 样本要求

直接测序法要求样本为肿瘤组织^[13, 23]^，是因为直接测序法的敏感性不高，不宜用于肿瘤组织外其它样本。Guo^[6]^等对此解释为非组织样本中浓缩提取的DNA难以达到分光光度法所能检测的最低限度，从而导致小剂量低比例的肿瘤衍生DNA被直接测序法误判为EGFR突变野生型。许多研究^[14, 19, 21, 30]^都证实了这一说法。其它方法中PCR-SSCP法和PCR-RFLP法仅见到用于肿瘤组织检测，未见有在其它标本中检测介绍。PCR-TaqMan法、DHPLC法、SARMS法、酶切富集PCR法均有肿瘤组织标本外样本相关研究介绍。其中DHPLC法、SARMS法和酶切富集PCR法被用于比较配对肿瘤组织和外周血样本EGFR突变比较的研究。

### 2.2 检测结果

#### 2.2.1 肿瘤组织

肿瘤组织样本中上述方法均与直接测序法进行过配对比较，PCR-TaqMan法与直接测序法的检测一致率达到95%以上^[8, 9, 31]^，而且PCR-TaqMan法敏感性在个别报道中还略高于直接测序法，白桦等^[10]^研究了230例晚期NSCLC患者外周血和肿瘤组织配对样本，结果显示肿瘤组织中DHPLC法与直接测序法相比灵敏度与特异度分别为96.9%和91.9%。陈思远等^[11]^也进行了类似研究，证实了两种方法中DHPLC法灵敏度为100%，特异度为95.31%，阳性一致率为86.36%。张静等^[18]^采用SARMS法和直接测序法检测了82例NSCLC组织*EGFR*突变情况，结果SARMS法检测到了82例中42例存在*EGFR*突变，突变率为51.2%，PCR后直接测序仅获得58例可分析的测序结果，其中25例出现突变，突变率为30.5%，而且直接测序检测出的25例突变模式与SARMS法检测结果相一致。Horiike等^[19]^利用支气管穿刺得到的样本对比了上述两种方法，结果与张静等^[18]^的研究结论一致，都显示出SARMS方法比直接测序法敏感优越。Marchetti等^[16]^入组了860例NSCLC患者的肿瘤组织，采用PCR-直接测序法和PCR-SSCP法进行检测，其中454例鳞癌和31例大细胞癌未见到*EGFR*突变，375例腺癌中PCR-SSCP法检测到39例出现突变，突变率达10%，低于正常突变率25%-35%，可能原因与入组患者特点有关，而其中21%的突变经直接测序法未能检测到，与测序法相比该方法敏感性高，可以发现测序未能发现的突变。Asano等^[21]^比较了酶切富集PCR法和直接测序法在手术切除的肿瘤组织、CT引导下穿刺得到的肺组织和胸水这三种样本中检测的一致性情况，结果显示手术切除的组织中两种方法一致性最好，其它两种样本中酶切法远高于直接测序法。

#### 2.2.2 配对肿瘤组织和外周血样本

白桦等^[10]^采用DHPLC法对230例晚期NSCLC患者外周血和肿瘤组织配对样本进行突变检测，结果显示外周血与肿瘤组织突变阳性检测一致率为79.7%。Kimura等^[15]^应用SARMS方法检测42例配对肿瘤组织和外周血突变情况，结果在两种样本中，突变一致率达到92.9%，可见SARMS方法具有高度敏感性。何臣等^[23]^报道了酶切富集法检测18例外周血和组织学配对样本，结果两种样本一致率为94.4%，阳性一致率为87.5%。

#### 2.2.3 配对胸腔积液和外周血样本

何臣等^[32]^也利用酶切法检测了30例胸腔积液中（15例血浆标本配对）EGFR突变情况，结果配对样本中阳性一致率为87.5%。

### 2.3 敏感性

直接测序法敏感性不低于30%突变拷贝数^[33, 34]^，PCR-TaqMan法不低于10%^[6]^、DHPLC法5%左右^[10, 35-37]^、SARMS法为1%^[15]^、PCR-SSCP法为10%^[33, 34]^、酶切富集法PCR法1%^[31]^，PCR-RFLP法1%^[24]^。

### 2.4 检测范围

EGFR突变主要表现在18-21位点，尤其是19、21外显子敏感突变和20外显子的耐药突变，上述方法中直接测序法在样本类型和样本量理想化的前提下检测范围最为广泛，可检测EGFR外显子18、19、20、21四个外显子处的29种体细胞突变^[1, 2]^。PCR-TaqMan法存在探针设计的局限性，未发现针对20外显子突变设计的探针，不能像直接测序法那样一目了然地发现EGFR外显子18-21位各种不同类型的突变，同时也存在假阴性的可能。DHPLC对外显子的检测类型同样具有选择性，由于EGFR 20外显子上游5个-6个位点有特殊碱基杂化而导致检测结果出现干扰峰的存在^[38]^，限制了此法的应用。SARMS是针对已知突变设计探针，可以检测权威研究机构得出的29种突变。PCR-SSCP法未检测到20外显子T790M相关耐药突变。因为缺乏20外显子的限制性内切酶，酶切富集法现阶段同样不能对20外显子T790M突变进行检测。PCR-RFLP法具备检测18-21位点热点突变的能力。

### 2.5 操作难易度

直接测序费用高，时间长。TaqMan-MGB实时PCR法操作简便、快速、灵敏，数小时内即可出结果。DHPLC法检测快速、简便、高通量而且价格便宜。SARMS法通过*EGFR*突变检测试剂盒（即EGFR Scorpion Kit；DxS Ltd.Manchester，英国），检测起来方便快捷。国内厂家采用英国DxS公司的蝎子引物法的技术平台，通过技术革新，建立了ADx特异扩增技术即环状特异引物+双环探针技术，并将其商品化为ADxTM *EGFR*基因突变检测试剂盒，在灵敏度（检测能力）、检测突变位点数上都和英国的DxS Ltd.Manchester试剂盒保持了完全一致，但在试剂操作上国产试剂盒采用一步加样远比进口的预混分装方便快捷（数据来源于上海胸科医院09年11月结果）。PCR-SSCP法电泳时间较长，操作步骤比较繁琐，并且只能进行定性分析，操作时间比较长。酶切富集法步骤较繁琐，适于实验室研究，不适于临床检测。PCR-RFLP法相对其它方法所需仪器设备比较简单，花费较少。

## 3 小结与展望

通过对上述方法的详细分析，从检测样本角度看，当取得肿瘤组织时，直接测序法依然是当前*EGFR*突变检测的金标准^[8, 9]^。但由于其灵敏度低（只能检测到30%突变拷贝数）^[33, 34]^，所以当没有获取组织样本时，不推荐使用PCR-直接测序法进行*EGFR*突变检测。PCR-TaqMan法、DHPLC法、SARMS法、PCR-SSCP法、酶切富集PCR法，PCR-RFLP法在肿瘤组织中的检测结果和直接测序法都保持了较高的一致性，其中SARMS法、DHPLC法、PCR-SSCP法在一定程度上比直接测序法更加敏感。

外周血检测因其创伤小、操作简便，容易获取，正成为近几年研究的热点。研究^[39]^结果表明血浆游离DNA主要来自凋亡坏死的癌细胞，其遗传特性与肿瘤细胞基因组DNA相同，肿瘤患者血浆中存在大量游离DNA，含量约为健康人的10倍以上。但外周血标本的获取时间窗、获取后的贮存时间^[40]^、检测方法的选择以及肿瘤组织的异质性等依然是影响其与肿瘤组织标本突变情况的重要因素。

上述方法中PCR-TaqMan法、DHPLC法、SARMS法、酶切富集法PCR法均有对外周血检测的报道，DH-PLC法、SARMS法和酶切富集法PCR法被用于比较配对肿瘤组织和外周血样本EGFR突变研究，结果三种方法在两种标本中的阳性一致率均达到70%以上。

敏感性上SARMS法、酶切富集法PCR法、PCR-RFLP法都可以达到1%，而且SARMS法以及PCR-RFLP法的检测范围也可以同直接测序法在一定程度上相媲美。操作上以试剂盒最为方便快捷，同时适于推广到临床。

获得组织的情况下直接测序法为首选，同时可采用敏感性较高的SARMS法（ADxTM）或酶切富集PCR法以及PCR-RFLP法进行对比，进一步确定上述方法的敏感性和精确度。若对20外显子有突变检测要求时，只能采用SARMS（ADxTM）法或PCR-RFLP法进行比较。若脱离实验室在临床检测时只能选择SARMS法（ADxTM），但国产ADxTM操作起来更加方便，结果判定较为简便，用起来也更加经济。针对晚期不能手术的NSCLC患者的组织标本很难获得的现状，当得不到组织样本时，直接测序法被排除。可供选择的灵敏度高的方法有PCR-TaqMan法、DHPLC法、SARMS法（ADxTM）、酶切富集PCR法。同组织标本选择条件相似，若对20外显子有突变检测要求时只能采用SARMS（ADxTM），若脱离实验室时选择方法同组织样本。

PCR-SSCP法、PCR-RFLP法目前只有组织标本的相关研究，还需要临床其它肺癌样本研究的对照和支持。PNA-LNA以及LightCycle-PCR法因为临床检测*EGFR*突变研究较少，仍需进一步研究。

*EGFR*突变检测方法的研究会随着*EGFR*突变位点的不断深入研究而完善，在此基础上，结合临床实际情况，有目的地开展上述方法的大规模多中心的临床试验，为临床提供方便快捷筛选EGFR-TKI优势患者的方法具有重要的意义。
